# The desert tortoise trichotomy: Mexico hosts a third, new sister-species of tortoise in the *Gopherus morafkai*–*G. agassizii* group

**DOI:** 10.3897/zookeys.562.6124

**Published:** 2016-02-10

**Authors:** Taylor Edwards, Alice E. Karl, Mercy Vaughn, Philip C. Rosen, Cristina Meléndez Torres, Robert W. Murphy

**Affiliations:** 1 School of Natural Resources and the Environment. The University of Arizona, Tucson, AZ 85721 USA; 2 University of Arizona Genetics Core, University of Arizona, Tucson, AZ 85721 USA; 3 P.O. Box 74006, Davis, CA 95617 USA; 4 Paso Robles, CA 93446 USA; 5 Comisión de Ecología y Desarrollo Sustentable del Estado de Sonora, Sonora, México; 6 Royal Ontario Museum, Toronto M5S 2C6, Canada

**Keywords:** Gopherus agassizii, Gopherus morafkai, Sinaloa, Sonora, Testudinidae, Xerobates

## Abstract

Desert tortoises (Testudines; Testudinidae; *Gopherus agassizii* group) have an extensive distribution throughout the Mojave, Colorado, and Sonoran desert regions. Not surprisingly, they exhibit a tremendous amount of ecological, behavioral, morphological and genetic variation. *Gopherus agassizii* was considered a single species for almost 150 years but recently the species was split into the nominate form and Morafka’s desert tortoise, *Gopherus morafkai*, the latter occurring south and east of the Colorado River. Whereas a large body of literature focuses on tortoises in the United States, a dearth of investigations exists for Mexican animals. Notwithstanding, Mexican populations of desert tortoises in the southern part of the range of *Gopherus morafkai* are distinct, particularly where the tortoises occur in tropical thornscrub and tropical deciduous forest. Recent studies have shed light on the ecology, morphology and genetics of these southern ‘desert’ tortoises. All evidence warrants recognition of this clade as a distinctive taxon and herein we describe it as *Gopherus evgoodei* **sp. n.** The description of the new species significantly reduces and limits the distribution of *Gopherus morafkai* to desertscrub habitat only. By contrast, *Gopherus evgoodei* **sp. n.** occurs in thornscrub and tropical deciduous forests only and this leaves it with the smallest range of the three sister species. We present conservation implications for the newly described *Gopherus evgoodei*, which already faces impending threats.

## Introduction

**Figure 1.**
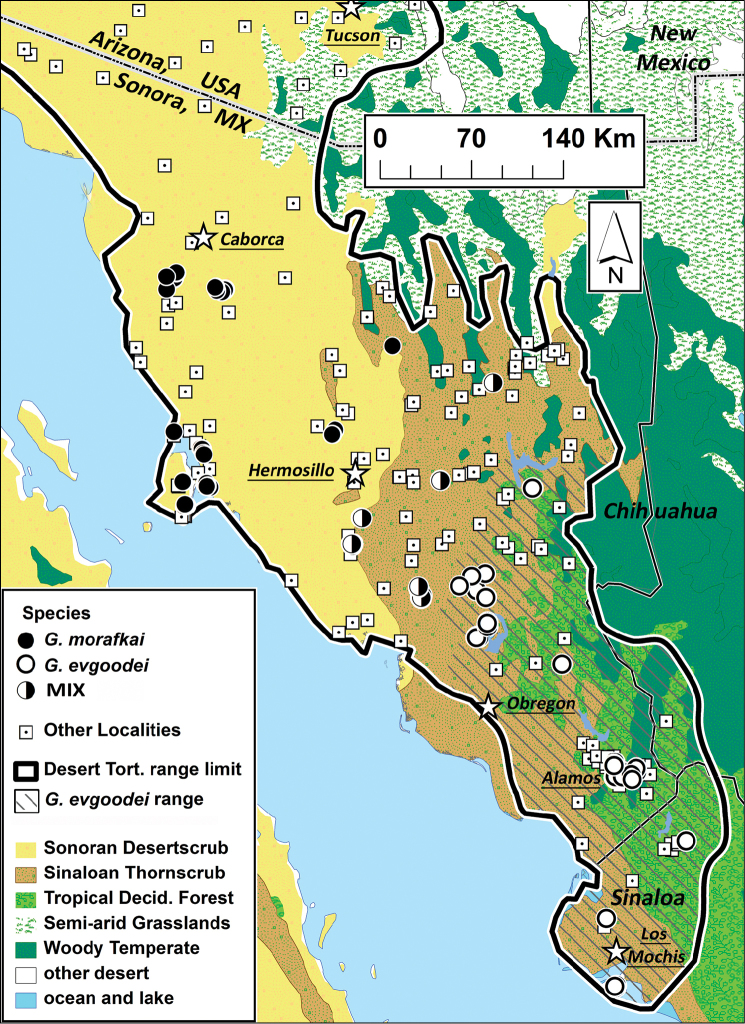
Conservative estimate of the distribution of *Gopherus evgoodei* in Mexico indicated by diagonal lines. Desert tortoise range limit, modified based on our field sampling, from Germano et al. (1994). Squares indicate museum and literature records of occurrence of *Gopherus* spp. Circles are sample locations from Edwards et al. (2015) for both *Gopherus morafkai* (black) and *Gopherus evgoodei* (white). Localities in the Sinaloan thornscrub-Sonoran desertscrub ecotone indicated by split circles, which indicate the occurrence of both *Gopherus evgoodei* and *Gopherus morafkai* genotypes and/or hybrids.

Desert tortoises (genus *Gopherus*: *Gopherus agassizii* group) occupy a large geographic range throughout the Mojave and Colorado deserts and in the Sonoran desert region of the United States and mainland Mexico (Fritts and Jennings 1994; Berry et al. 2002) (Figure 1). *Gopherus morafkai* (Murphy et al. 2011) was described as a species separate from *Gopherus agassizii* (Cooper 1861) based on ecological, behavioral and genetic differences. Murphy et al. (2011) noted that the full diversity of *Gopherus morafkai* had not yet been defined. Lamb et al. (1989) reported deeply divergent mitochondrial DNA (mtDNA) haplotypes in the southern portion of the range of *Gopherus morafkai*. Edwards et al. (2015) conducted a detailed genetic analysis of *Gopherus morafkai* in Mexico. They found that this southern “Sinaloan lineage” constituted a species distinct from northern congeners.

Ecological and morphological characteristics distinguish the northern “Sonoran” and southern “Sinaloan” lineages of tortoises found in thornscrub and tropical deciduous forest of southern Sonora and northern Sinaloa from those occurring further north in Sonoran Desertscrub (Bogert and Oliver 1945; Loomis and Geest 1964; Hardy and McDiarmid 1969; Germano 1993; Fritts and Jennings 1994; Berry et al. 2002; Bury et al. 2002; Legler and Vogt 2013). The three biomes, including Sonoran Desertscrub (SDS), Sinaloan Thornscrub (STS), and Tropical Deciduous Forest (TDF), occur in the area known broadly as the Sonoran Desert region (*sensu* Brown et al. 1979; Brown 1994; Martin et al. 1998). Whereas the Sonoran lineage of *Gopherus morafkai* ranges throughout SDS in Sonora, Mexico and Arizona, USA, the Sinaloan lineage occurs solely in TDF and STS environments (Figure 1; Edwards et al. 2015). The two lineages occur sympatrically in a relatively narrow ecotone between the SDS and STS and limited hybridization occurs only in this region. No obvious geographic barrier limits introgression yet the two groups of tortoises are deeply diverged genetically and they maintain their unique identities (Edwards et al. 2015, 2016). Because the Sinaloan lineage is genetically, ecologically and morphologically distinct from its congeners, below we describe it as a new species.

## Materials and methods

### Genetics

Edwards et al. (2015) assessed the population genetic structure of desert tortoises in the Sonoran Desert region by sampling 233 wild desert tortoises consisting of both Sonoran and Sinaloan lineages of *Gopherus morafkai*. They sampled their known distributions in each of the three major biomes where tortoises occur. They reconstructed matrilineal relationships using mtDNA sequences and employed 25 microsatellites (STRs) to perform Bayesian analyses of gene flow. They also conducted clinal analyses using both mtDNA and STRs to determine the position and amount of introgression where lineages co-occur. Further, Edwards et al. (2016) used mtDNA and four nDNA loci to perform a multi-locus phylogenetic analysis to estimate the species-tree among desert tortoise lineages. They also tested for ancestral lineage admixture with RNA-seq data based on diffusion approximation for demographic inference using the software package ∂A∂I (Gutenkunst et al. 2009).

Herein, to assist direct comparison with previous data, we add mtDNA divergence estimates for cytochrome *b* (*Cytb*) as well as the standard barcoding locus cytochrome oxidase subunit I (COI) for species discrimination within the Cold Code project (Murphy et al. 2013). For *Cytb*, we used primers H16464 and L14724 to amplify an approximately 1,500 bp fragment following the methods developed for *Gopherus* by Osentoski and Lamb (1995). We generated sequences across the entire amplicon by sequencing with both the amplification primers and with internal sequencing primers CytbF2 and CRR3 developed by Clostio et al. (2012). We then aligned these sequences to available sequences in GenBank for *Gopherus agassizii* (Accession AY434562.1) to generate a 1,140 bp sequence for each sample. For *COI*, we used primers L-turtCOIc and H-turtCOIc and followed protocols developed by Stuart and Parham (2004). For both loci, we generated sequences for *Gopherus agassizii* (n = 4; sampled from Nevada and California), Sonoran lineage of *Gopherus morafkai* (n = 4; sampled from Arizona, USA and Sonora, MX) and the Sinaloan lineage of *Gopherus morafkai* (n = 2; from Sonora and Sinaloa, Mexico). In addition, we included their closet outgroup based on Lamb et al. (1989), *Gopherus berlandieri* (Agassiz) (n = 2), for comparison. We estimated divergence among the species of *Gopherus* using DNASP (v.5.10.01; Librado and Rozas 2009).

### Morphology

Bogert and Oliver (1945) first recognized the distinct morphology of the southern, Sinaloan lineage of *Gopherus morafkai*, but they were unable to quantify it due to very small sample sizes. Other studies have also noted morphological characteristics that distinguish the Sinaloan lineage but did not provide a quantitative analysis (Loomis and Geest 1964; Hardy and McDiarmid 1969; Germano 1993; Fritts and Jennings 1994; Berry et al. 2002; Bury et al. 2002; Legler and Vogt 2013). We observed distinct morphological characters in 23 tortoises in the vicinity of Alamos, Sonora in 2005. To this, we added anecdotal observations and measurements of several preserved specimens of Sonoran and Sinaloan lineages of *Gopherus morafkai* and *Gopherus agassizii* in the University of Arizona herpetological collection and data from McLuckie et al. (1999). Consequently, we developed a suite of measurements and qualitative factors that morphologically diagnose the Sinaloan lineage. Measurements (in mm) included the following 37 variables: mid-carapace length (MCL); maximum width; maximum width at 3/4 marginal scute seam; maximum width at mid-6th marginal; maximum width at 7/8 marginal scute seam; width of C-truss 1 (left); width between anal tips; rear foot-pad greatest width; maximum height; height at 2nd vertebral scute; height at 3rd vertebral scute; height at 4th vertebral scute; maximum plastron length from tip of gular horn to tip of anal scutes; length of plastron truss (left); length of plastron shortest diagonal; length of right pectoral scute; length of left pectoral scute; average midline length of abdominal scutes; average midline length of femoral scutes; average midline length of anal scutes; depth of male concavity; distance of posterior shell opening from anal tip to carapace; distance of supracaudal scute to anal notch; distance of mid-9th marginal to inner femoral; distance of mid-9th marginal to outer femoral; distance of anterior shell opening; distance of nuchal to plastron; distance of mid-2nd marginal to humeral (inner and outer); distance of gular straight-line length; distance of curved length; head length from tip of rostrum to anterior corner of the eye; width of tympanum; height of bridge from 6th marginal to abdominal scutes; shortest bridge length; distance of anterior bridge opening to inner 2nd/3rd marginal scute seam; distance of anterior bridge opening to outer 2nd/3rd marginal scute seam; and distance from bridge to inguinal point of attachment. We took straight-line measurements only. We also assessed the following 13 qualitative characters: shape of rear feet (flat/rounded); presence of spur at humeral junction; shape of anterior and posterior armoring scales (rounded/pointed); spikiness of rear and front legs (high/moderate/low); lateral profile of shell (flat/domed); profile of pre-frontals; wear-class of shell; carapace color; plastron color; integument color; annuli distinct (yes/no); and tail length (shorter or same as *Gopherus agassizii*).

Measurements were taken on most tortoises that we encountered during field trips in Sonora and northern Sinaloa from 2006 through 2012. These animals served as the genetic resources for Edwards et al. (2015). From this larger dataset, we analyzed a subset of tortoises whose genetic lineage was verified using molecular diagnostics, including 62 individuals of Sonoran (*Gopherus morafkai*; n=16) and Sinaloan lineages (n=36) sampled in Mexico, as well as 10 tortoises of mixed lineage. We compared these tortoises to three populations of *Gopherus agassizii* in the Mojave and Colorado deserts of California (n=109). Populations near Algodones Dunes in eastern Imperial County (n=19) and near California City in eastern Kern County (n=64) were at the southeastern and northwestern geographic limits of the range of *Gopherus agassizii* in California, respectively. A third population from 55 km southeast of Barstow, San Bernardino County (n=26) was from central Mojave Desert in California.

A comprehensive analysis of morphological characters for these desert tortoises does not exist. Thus, our taxonomic evaluation was based on a statistical analysis of the following variables that appeared to us to consistently diagnose the species by exhibiting little intraspecific variation: shell color; integument color; tail length; depth of male plastron concavity; presence of a spur at the radial-humeral joint; and roundness of carapace (e.g. dome-shape vs. flat) based on the ratio of the height at the 3rd vertebral scute to carapace length, while accounting for depth of plastron concavity in males. Shell and integument highlights and hues were coded based on the following wavelengths of colors (Encycolorpedia 2015): orange, 605 nm; yellow, 580 nm; olive, 570 nm; and brown and grey, 539 nm. These six variables were compared between groups that consisted of lineage/location using the ANOVA function in SYSTAT ver. 13 (Systat Software, Inc., San Jose, California). Analyses accounted for interactions by size and sex. Tukey’s post-hoc pairwise comparison was used to identify among-site differences.

### Area of occurrence

We estimated the area of occurrence for the Sinaloan lineage by using the web-based tool GEOCAT (http://geocat.kew.org/what). Due to having few data points and a hybrid zone, we did not calculate the area of occupancy. Estimated values for *Gopherus morafkai* and *Gopherus agassizii* were taken from U.S. Fish and Wildlife Service (2015).

## Results

Edwards et al. (2015) estimated a 5.7 Ma divergence between matrilines of *Gopherus morafkai*. Strong genetic differentiation occurred across the STR loci. Analyses indicated that *Gopherus morafkai* consisted of two genetically and geographically distinct species (referred to as “Sonoran” and “Sinaloan” lineages). Both lineages occurred in a relatively narrow zone of overlap in STS where limited introgression occurred (Fig. 1). Bimodal genetic clines for both mtDNA and nDNA coincided with ecological features where the lineages came into contact. Clinal analysis revealed a strong coincidence of slope and concordance of centers for the mtDNA and nDNA markers. These occurrences dismissed cytonuclear discordance as an explanation for the observations (Toews and Brelsford 2012). The shifting ecotone between STS and SDS biomes may have acted as an ephemeral boundary that fostered adaptations in each lineage, and resulted in a largely parapatric distribution. Despite incomplete reproductive isolation, the two lineages of *Gopherus morafkai* maintained separate evolutionary trajectories.

Edwards et al. (2016) presented a species-tree reconstructed using a multi-locus Bayesian species delimitation analysis reconstructed from mtDNA and four nDNA loci. The tree depicted Sonoran and Sinaloan tortoises as sister lineages and together they formed the sister to *Gopherus agassizii*. Nodes of the tree had overlapping standard deviations. This tree topology was consistent with that of an independent analysis of 15 nuclear loci performed by Spinks et al. (P. Spinks, University of California Los Angeles; *personal communication*). In their RNA-seq analysis, Edwards et al. (2016) also characterized 20,126 synonymous variants from 7,665 contigs in six individuals, two representing each of the three lineages. The best-fit model observed from the ∂A∂I analysis was concordant with their multilocus species tree but more clearly elucidated the relative divergence times among the lineages. This result suggested that the Sonoran/Sinaloan split occurred only a short time after (or possibly even simultaneous with) divergence of *Gopherus agassizii*. Thus, the three lineages formed a trichotomy with relatively equal levels of divergence from each other. The ∂A∂I analysis also failed to detect evidence of gene flow during divergence among the three lineages. Analyses revealed that divergence among the lineages occurred in the absence of gene flow, whether through physical allopatry or ecological niche segregation. The results further validated species-level differentiation among the three lineages.

### MtDNA sequence divergence

We generated a 761 bp sequence of mtDNA that encodes part of the gene encoding *COI* and identified seven unique haplotypes in our sample set (GenBank accession numbers; KR610436–KR610442). Divergence at *COI* between *Gopherus agassizii* and Sonoran *Gopherus morafkai* was 4.1%, between Sinaloan lineage tortoises and *Gopherus agassizii* 3.6%, and between Sinaloan lineage tortoises and Sonoran *Gopherus morafkai* 3.4%. Divergence between all three species/lineages of desert tortoise with *Gopherus berlandieri* averaged 6.1%. For *Cytb*, we generated 1140 bp sequences and identified six haplotypes (GenBank Accession No. KT956833–KT956838). We included GenBank sequences from *Gopherus agassizii* (Accession No. AY434562.1) in our alignment and analyses. Divergence at *Cytb* between *Gopherus agassizii* and Sonoran *Gopherus morafkai* was 4.5%, between Sinaloan lineage tortoises and *Gopherus agassizii* 3.7%, and between Sinaloan lineage tortoises and Sonoran *Gopherus morafkai* 4.2%. Divergence between all three species/lineages of desert tortoise with *Gopherus berlandieri* averaged 5.9%.

### Morphology

**Figure 2.**
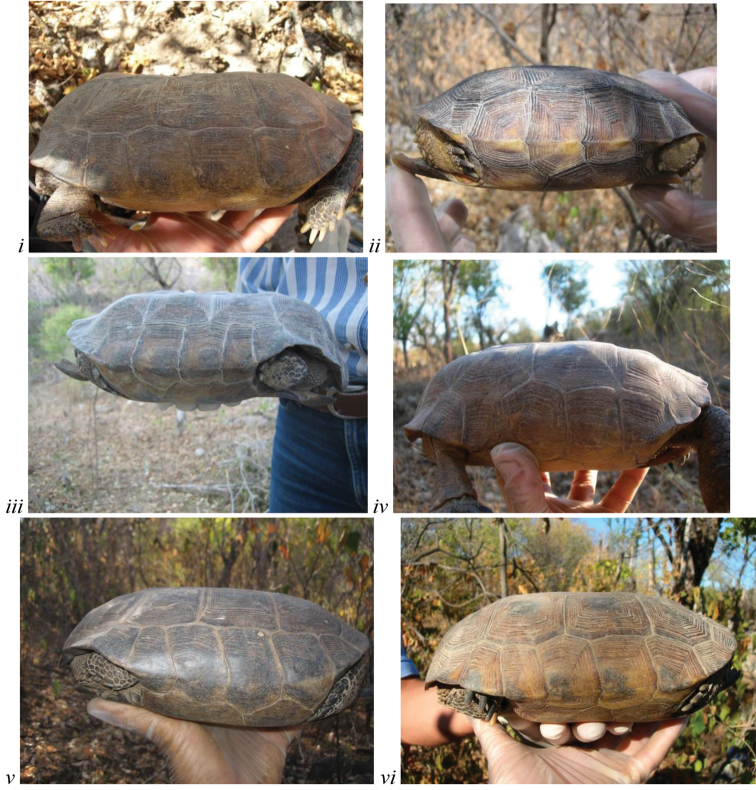
The flat shell profile/shape of carapace generally distinguishes *Gopherus evgoodei* from other species of desert tortoises. Live, wild-caught individuals from (**i–iv**) Rancho Las Cabras and (**v–vi**) Rancho La Sierrita near Alamos, Sonora, Mexico (in Tropical Deciduous Forest).

**Figure 3.**
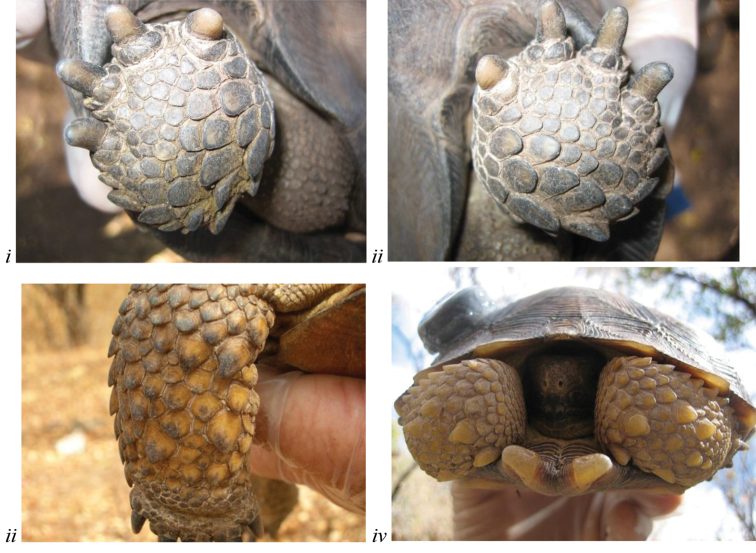
The rounded ventral surface of the rear feet (**i–ii**) and multiple enlarged, raised scales present on surface of forelegs generally (**iii–iv**) diagnose *Gopherus evgoodei* in relation to other species of desert tortoises. **i–ii** same individual in Figure 2 from Rancho Las Cabras near Alamos, Sonora, Mexico (in Tropical Deciduous Forest) **iii–iv** two individuals from Rancho Las Cabras.

All species of *Gopherus* shared the following morphological characteristics with other members of the family Testudinidae (Ernst and Barbour 1989): 11 marginal scutes on both right and left edges of the carapace; five toenails on each forelimb and four toenails on each elephantine hind limb. Within the desert tortoises and like *Gopherus agassizii* and *Gopherus morafkai*, the Sinaloan lineage tortoise was sexually dimorphic with mature males having a slightly longer tail, enlarged gular horn, a concave plastron, a tucked supracaudal scute and prominent chin glands. However, several characteristics generally distinguished the Sinaloan lineage from other desert tortoises. Sinaloan tortoises had a very flat carapace (Fig. 2) that was highly significantly flatter than the conspicuously domed carapaces of *Gopherus agassizii* and Mexican *Gopherus morafkai* (F_5,162_ = 6.789; p<0.0005). All Sinaloan tortoises (100% of 37 adults) had prominent, pointed scale(s) (spurs) at the humeral/radial joint (Fig. 3). *Gopherus morafkai* and *Gopherus agassizii* also had spurs, but less consistently, and they were rarely prominent or pointed in *Gopherus agassizii*. Only 25% of Mexican *Gopherus morafkai* (n=16) and 15.9% of *Gopherus agassizii* from the Colorado Desert had spurs (n=19). Interestingly, 73.9–74.6% of the tortoises (n=86) from the Mojave Desert had spurs. There were too few small tortoises to detect an association between size and presence of spurs. The occurrences of spurs did not differ between sexes.

**Figure 4.**
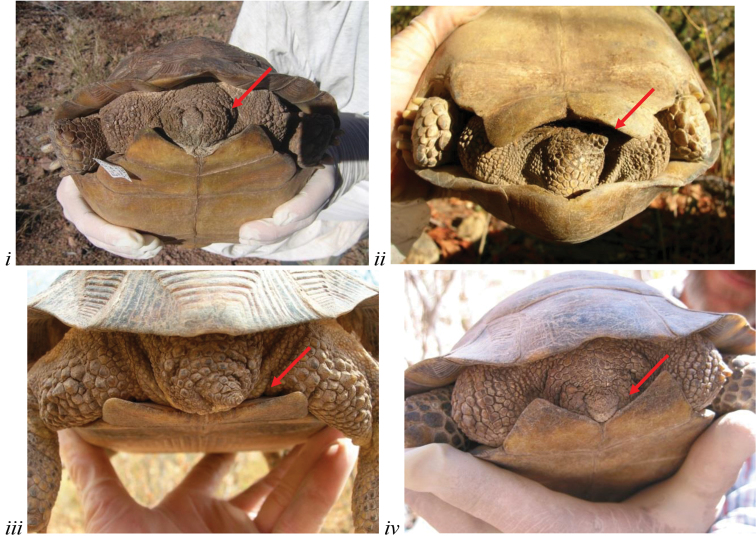
*Gopherus evgoodei* differs from other species of desert tortoises in having a very short tail. **i** Rancho El Chupadero east of Guaymas (in thornscrub habitat); **ii** Rancho Las Cabras; and **iii–i** Rancho La Sierrita near Alamos, Sonora, Mexico (in Tropical Deciduous Forest).

While male *Gopherus* have longer tails than females in all species, Sinaloan lineage tortoises differed highly significantly from the other desert tortoises in having a very short tail in both sexes (F_5,153_ = 56.044; p<0.0005) (Fig. 4). The tails of female Sinaloan tortoises were frequently little more than nubs (2–8 mm) and those of males and female *Gopherus agassizii* were the same size (<13 mm).

**Figure 5.**
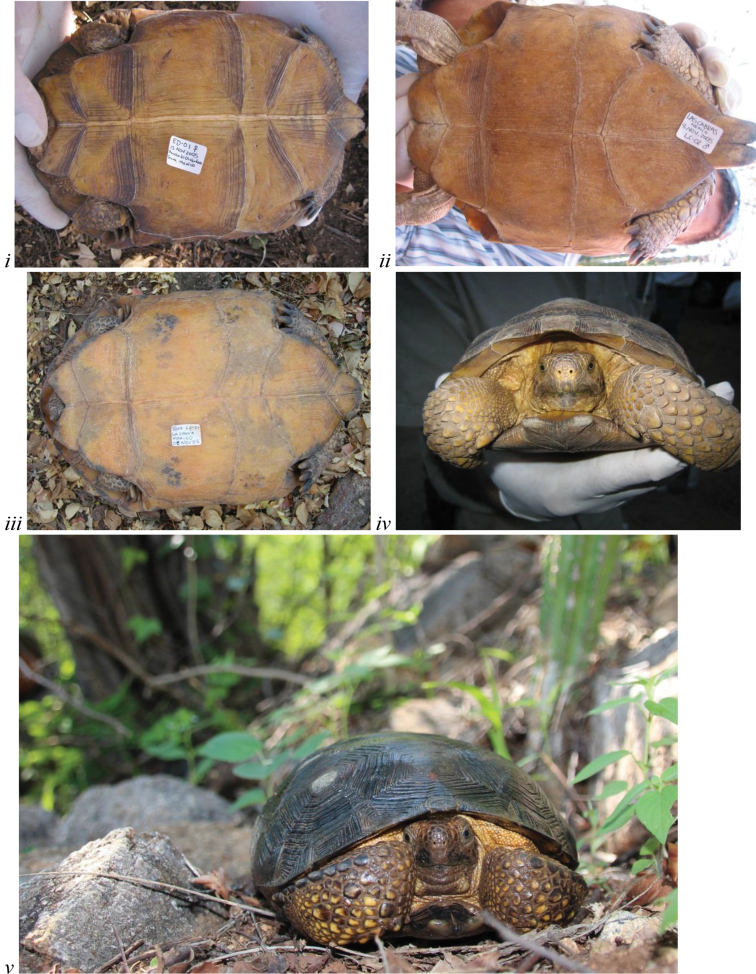
*Gopherus evgoodei* differs from other species of desert tortoises in often having yellow/orange integument (skin) and shell. **i** Rancho El Divisadero **ii–iii** Rancho Las Cabras; and **iv–v** Rancho La Sierrita near Alamos, Sonora, Mexico (in Tropical Deciduous Forest).

Subdued shell mottling and spotting differed highly significantly in Sinaloan lineage tortoises (orange hues) versus *Gopherus agassizii* and Sonoran *Gopherus morafkai* (F_5,162_ = 49.118; p<0.0005) (Fig. 5); the shells of both *Gopherus morafkai* and *Gopherus agassizii* were medium to dark brown or dark gray, sometimes with a subtle greenish hue and generally dark gray to dark brownish-gray near scute interfaces. The integument of *Gopherus morafkai* and *Gopherus agassizii* tortoises was dark gray to brownish-gray and this differed highly significantly from the dark tan to medium-brownish coloration, with a distinctly orange cast, in Sinaloan lineage tortoises (F_5,152_ = 58.137; p<0.0005).

**Figure 6.**
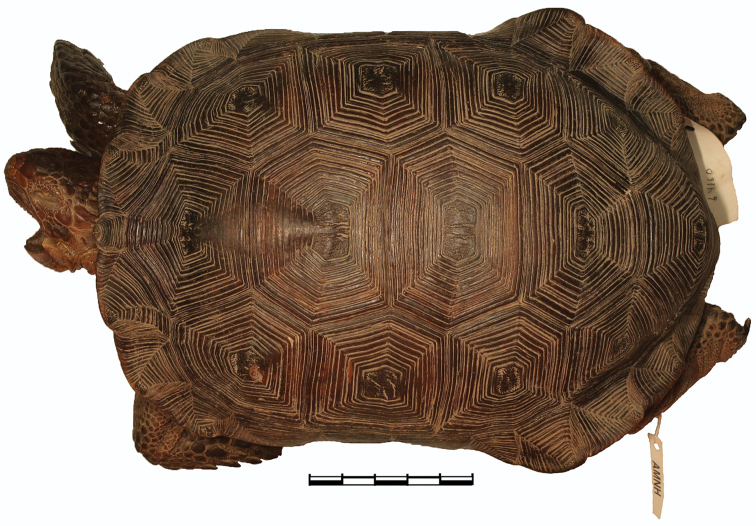
Dorsal view of the holotype of *Gopherus evgoodei*, AMNH R64160. Scale bar 50 mm in 10 mm increments.

The concavity on the plastron of male Sinaloan lineage tortoises was similar to that of *Gopherus morafkai* yet highly significantly shallower than that of *Gopherus agassizii* (F_5,77_ = 17.885; p<0.0005). Several other morphological characters appeared to consistently diagnose the Sinaloan lineage tortoises. Sinaloan lineage tortoises typically displayed rounded pads on the rear feet (Fig. 3) while the pads of *Gopherus agassizii* in the northern Mojave Desert were generally flat. Whereas the Sinaloan lineage tortoises were distinctly bulbous over the pre-frontal scales in profile, *Gopherus agassizii* in the Sonoran Desert of California was generally rounded (Fig. 6), and Mexican *Gopherus morafkai* was flat to slightly round. The nictitating membrane of tortoises of the Sinaloan lineage was generally pink and enlarged. In *Gopherus morafkai*, and less so in *Gopherus agassizii*, the nictitans may have been enlarged but rarely pink, and, if pink, it indicated an inflammation (USFWS 2013).

### Area of occurrence

Analyses using GeoCAT suggested that the distribution of the Sinaloan lineage encompassed roughly 24,000 km^2^. We could not calculate the area of occupancy owing to the limited number of data points.

To varying degrees, some of these morphological differences have been recognized in other studies (Bogert and Oliver 1945; Loomis and Geest 1964; Hardy and McDiarmid 1969; Germano 1993; Fritts and Jennings 1994; Berry et al. 2002; Bury et al. 2002; Legler and Vogt 2013). The new genetic assessments (Edwards et al. 2015, 2016) and our morphological analyses and assessment of habitat preferences suggest that *Gopherus morafkai* is a composite of two species. As such, the current taxonomy may negatively affect efforts to conserve both species. Herewith, we describe the Sinaloan lineage of desert tortoise as a new species.

#### Gopherus evgoodei

Taxon classificationAnimaliaTestudinesTestudinidae

Edwards, Karl, Vaughn, Rosen, Meléndez Torres & Murphy sp. n.

http://zoobank.org/125138E1-31AC-4FE5-8971-2F3D0A5113B8

Goode’s Thornscrub Tortoise

Figs 6 , 7 , 8 , 9 , 10 , 11 , 12 , 13 , 14

Xerobates agassizii Cooper, 1861 (partim)
Gopherus agassizii (Cooper, 1861) (partim). Generic reassignment by Stejneger (1893)
Scaptochelys agassizii (Cooper 1861) (partim). Generic reassignment by Bramble (1982)
Xerobates lepidocephalus (ex errore) Ottley and Velázques Solis 1989. In error by Crumly and Grismer (1994)
Gopherus morafkai Murphy, Berry, Edwards, Leviton, Lathrop & Riedle, 2011 (partim)

##### Holotype.

AMNH (American Museum of Natural History) R64160; adult male from Alamos (approximate location 27°02'N, 108°55'W, elevation 433 m), Sonora, Mexico, collected on 27 August–2 September 1942 by Charles M. Bogert and preserved in ethanol (Figs 6–14).

**Figure 7.**
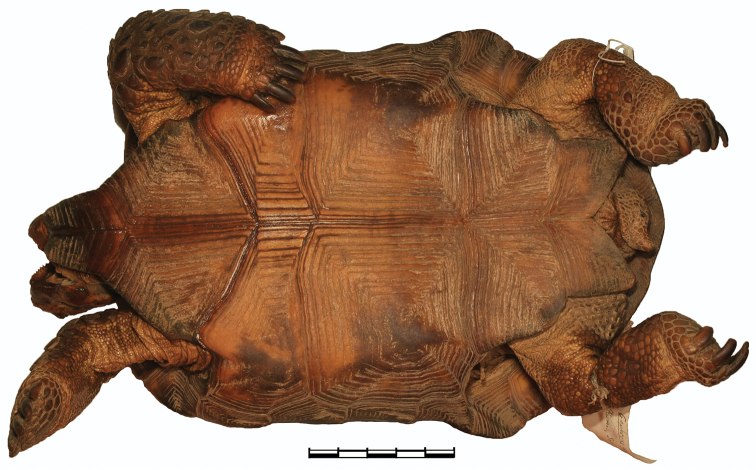
Ventral view of the holotype of *Gopherus evgoodei*, AMNH R64160. Scale bar 50 mm in 10 mm increments.

**Figure 8.**
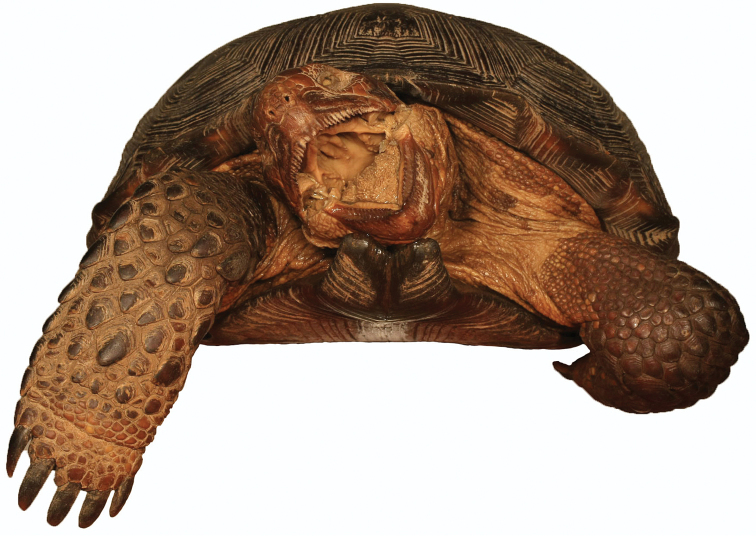
Anterior view of the holotype of *Gopherus evgoodei*, AMNH R64160.

**Figure 9.**
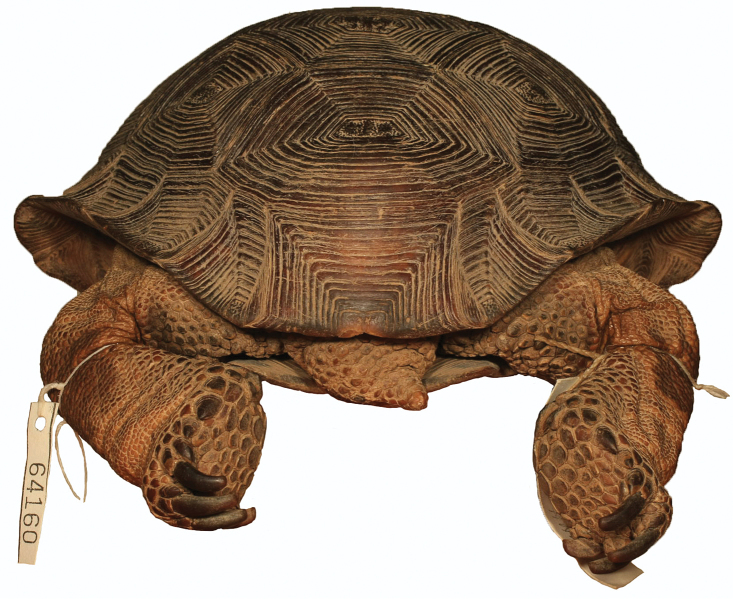
Posterior view of the holotype of *Gopherus evgoodei*, AMNH R64160.

**Figure 10.**
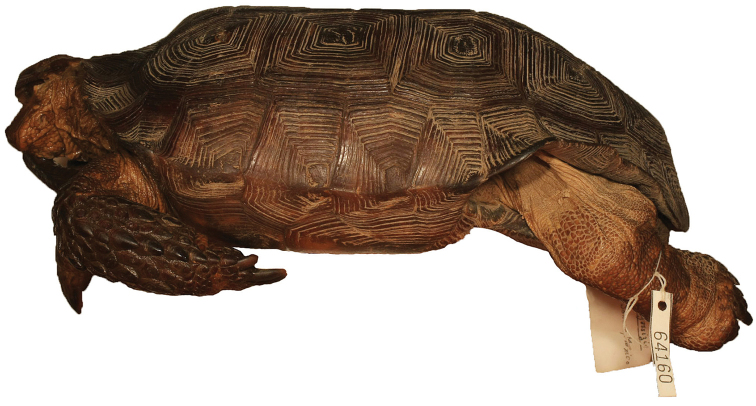
Left lateral view of the holotype of *Gopherus evgoodei*, AMNH R64160.

**Figure 11.**
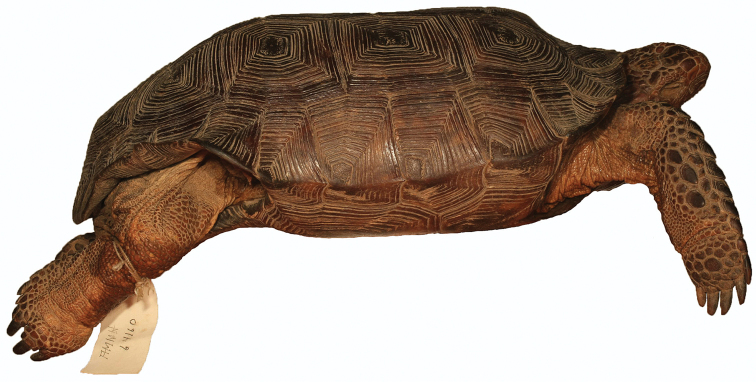
Right lateral view of the holotype of *Gopherus evgoodei*, AMNH R64160.

**Figure 12.**
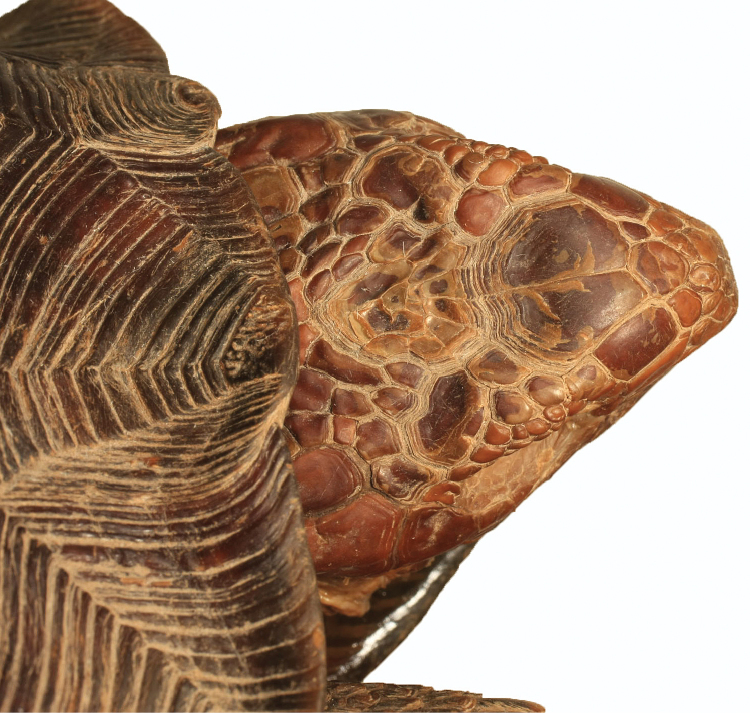
Detail of head scales of the holotype of *Gopherus evgoodei*, AMNH R64160.

**Figure 13.**
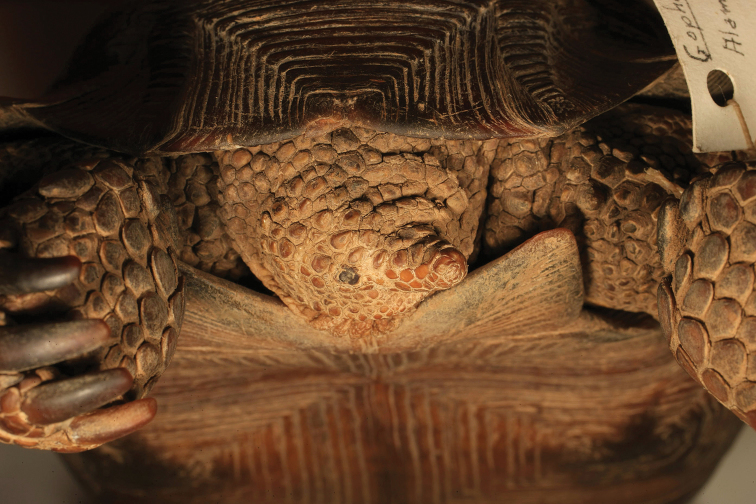
Detail of the tail of the holotype of *Gopherus evgoodei*, AMNH R64160.

**Figure 14.**
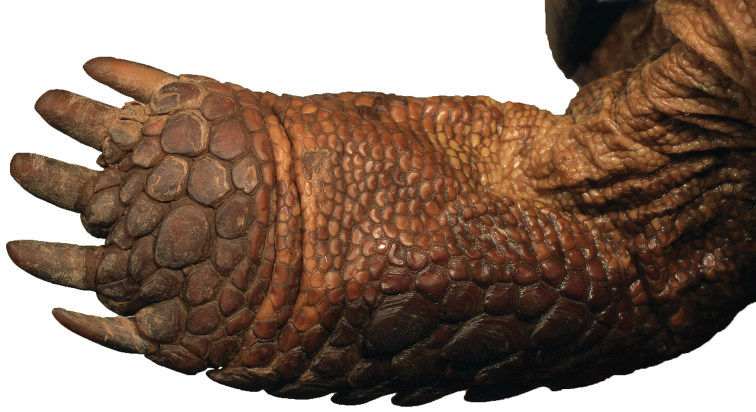
Ventral surface of the right rear foot of the holotype of *Gopherus evgoodei*, AMNH R64160.

##### Paratypes.

AMNH R64157, an adult male; AMNH R64158, an adult female; and ROM (Royal Ontario Museum) 53301 (formerly AMNH R64159), an adult female; all with same collecting data as the holotype and all preserved in ethanol.

##### Referred specimens.

ASU (Arizona State University, Tempe) 6427, ASU 6543–44, ASU 6605–06, ASU 6620–22, ASU 6702–03, ASU 6769, ASU 8534–39, CAS (California Academy of Sciences) 142243, CM (Carnegie Museum) Herps:62200, CNAR (Colección Nacional de Anfibios y Reptiles)-4002, LACM (Los Angeles County Museum) 105338, LSUMZ (Louisiana State University Museum of Zoology, Baton Rouge) 34925, MSB (Museum of Southwestern Biology) MSB 41497–99, MVZ (Museum of Vertebrate Zoology) 129943, SMNS (Staatliches Museum fuer Naturkunde, Stuttgart) 7367–68, SMNS 7515, TNHC (Texas Memorial Museum) 60607, UAZ (University of Arizona) 28105, UAZ 35405, UAZ 36875–76, UAZ 56589-PSV, UAZ 56607-PSV, and UIMNH (University of Illinois Museum of Natural History) 85836.

##### Diagnosis.

Molecular data can readily diagnose all species of *Gopherus* and their hybrids (Murphy et al. 2011; Edwards et al. 2016). Morphologically, *Gopherus evgoodei*, *Gopherus agassizii* and *Gopherus morafkai* (the *agassizii* group) can be separated generally from both *Gopherus flavomarginatus* Legler and *Gopherus polyphemus* (Daudin) in having relatively smaller front feet. Whereas the distance from the bases of the first to fourth claws is the same on all feet in the *agassizii* group, in the latter two species the distance from the bases of the first and third claws on the forelimb is about the same as the distance between the bases of the first and fourth claws on the hindlimb (Auffenberg and Franz 1978). Living captive specimens of the *agassizii* group and *Gopherus berlandieri* cannot all be distinguished morphologically because of extensive hybridization (Edwards et al. 2010) and developmental abnormalities in shell, head and limb integument from poor nutrition (Donoghue 2006). However, in native non-hybrid individuals, *Gopherus berlandieri* can be separated from the *agassizii* group in having a wedge-shaped snout when viewed from above in contrast to a rounded snout (Fig. 12) (Auffenberg and Franz 1978). Further, the gular projections of *Gopherus berlandieri* often diverge in large males and the species often exhibits paired axillary scales preceding each bridge. In contrast, the gular projections do not normally diverge in the *agassizii* group and there is a single axillary scale. Morphological characters among the *agassizii* group exhibit overlap (Germano 1993; McLuckie et al. 1999) and characters like coloration in desert tortoises can be highly variable (Legler and Vogt 2013). However, *Gopherus evgoodei* differs from *Gopherus morafkai* and *Gopherus agassizii* (Table 1). *Gopherus evgoodei* is flatter in shell profile (Fig. 2). It has rounded foot pads, multiple enlarged spurs on the radial-humeral joint (Fig. 3). The new species has short tails (Fig. 4), orange tones in the integument (skin) and shell (Fig. 5), and a distinctly shallower concavity on the plastron of males.

**Table 1.** Least-square means (LSM) and sample size (N) for ANOVA for five morphometric characters that are highly descriptive for *Gopherus evgoodei* and frequency percentages for one character. Mixed samples from localities in the Sinaloan thornscrub-Sonoran desertscrub ecotone with the occurrence of both *Gopherus evgoodei* and *Gopherus morafkai* genotypes and/or hybrids. Carapace shape measures ‘roundness’ of carapace.

	Variable											
Lineage (Location)	Shell Color		Integument color		Tail length		Male plastron concavity		Carapace shape		Humeral spurs	
	LSM	N	LSM	N	LSM	N	LSM	N	LSM	N	% with spurs	N
*Gopherus evgoodei* (Mexico)	601.686	35	593.543	35	0.61	33	10.185	17	0.419	36	100.0	37
Mixed *Gopherus evgoodei* / *Gopherus morafkai* (Mexico)	574.8	10	560.667	9	0.778	9	13.8	4	0.447	10	62.5	8
*Gopherus morafkai* (Mexico)	548.25	16	541.563	16	0.833	12	11.35	8	0.454	16	25.0	16
*Gopherus agassizii* (Imperial County, California)	562.706	17	546.882	17	0.947	19	25.312	10	0.449	18	15.8	19
*Gopherus agassizii* (San Bernardino County, California)	549.654	26	552.882	17	0.96	25	19.786	8	0.461	25	73.9	23
*Gopherus agassizii* (Kern County, California)	563.219	64	543.578	64	0.934	61	22.844	36	0.454	63	74.6	63

##### Description of holotype

(parallels that of *Gopherus morafkai* by Murphy et al. 2011). An adult male, with carapace length at the midline (MCL) = 209 mm; curved carapace length from nuchal scute to supracaudal scute = 254 mm; plastron from tip of gular horn to tip of anal scutes = 219 mm; plastron from gular notch to anal notch = 202 mm; maximum height of shell at 3rd vertebral scute = 83 mm; width at 3rd/4th marginal scute seam = 137 mm; width at 6th marginal scute = 140 mm; greatest width at mid-8th marginal scute = 158 mm; plastron concavity depth = 10.1 mm; head length = 51.3 mm; and tail = 8 mm. Eleven marginal scutes present on both right and left edges of the carapace. Five toenails present on each forelimb and four toenails on each hind limb. The third nail of each hind limb slightly longer than the others. Multiple enlarged, raised scales present on the anterior ventral surface of each foreleg. No scale “spikiness” on the posterior femoral surface of the rear legs. Scales on head smooth and asymmetrical, with two large pre-frontal scales and smaller scales in the temporal area. Shape of head prefrontal profile rounded/bulging. Shell profile/shape of the carapace appearing nearly flat. Shape of ventral surface of rear feet rounded and lacking projecting, enlarged scales on the posterior plantar surface. Areolae and >17 growth laminae present on all carapacial scutes, although areola are diminishing, especially on the anterior scutes. In alcohol, the color of areolae dark, fading to dark brown with orange hue in outer portion of carapacial scutes. Color of areolae on the plastron dark brown and rest of the plastron medium orange brown. Head and neck tan to dark tan with an orange hue. Skin in the axillary and inguinal areas lighter in coloration; light tan fading to medium tan toward axillary. Nails dark brown, lighter brown at the tips.

##### Coloration of the species in life.

*Gopherus evgoodei* may exhibit orange or yellow mottling or spotting on the shell and integument. Because color constitutes a diagnostic feature, these data are given above.

##### Variation.

As with all species of *Gopherus*, substantial variability exists among individuals for most morphological features (Germano 1993; McLuckie et al. 1999). Bogert and Oliver (1945) first recognized the distinct morphology of tortoises at Alamos, but they were unable to quantify it due to small sample sizes. Shell profile is generally flat but may also appear domed in some individuals. Spikiness of scales on forelimbs can vary widely and the shape of the plantar surface of the rear feet, while generally rounded, can be difficult to classify in some cases.

##### Distribution.

The distribution of *Gopherus evgoodei* (Fig. 1) occupies roughly 24,000 km2 and corresponds to habitat. The species primarily occurs in tropical deciduous forest (TDF) and relatively mesic Sinaloan thornscrub (STS) in the state of Sonora, Mexico, and its distribution extends southward into TDF and the southern part of the STS where it still remains intact in northern Sinaloa south of the Río Fuerte (Loomis and Geest 1964; Edwards et al. 2015). It also occurs in the TDF of extreme southwestern Chihuahua (Smith et al. 2004). Thus, *Gopherus evgoodei* occupies both STS and Sinaloan TDF (Fritts and Jennings 1994; Berry et al. 2002). The eastern limit of its known range is the foothills of the Sierra Madre Occidental at elevations of 800–1,000 m where the TDF transitions rather abruptly into oak woodlands (Bury et al. 2002). Although the southern limit of its range remains undetermined, continuous TDF extends along the West Coast of Mexico from Sonora through Sinaloa to Nayarit (>500 km), although it only maintains an average width of 50 km (Krizman 1972). It is unlikely that *Gopherus evgoodei* occurs very much further south in Sinaloa than currently known, or as far south as Nayarit, based on an absence of records for this relatively conspicuous and readily recognizable animal, and presumably due to as yet unidentified environmental limitations (Bury et al. 2002). The northern boundary of *Gopherus evgoodei* corresponds approximately to the transition from STS to SDS (Edwards et al. 2015). Although characteristic thornscrub maintains 100% ground cover, where it grades into desertscrub it becomes patchy (Felger et al. 2001). The transition of TDF and thornscrub to desertscrub dominated by more xeric species often occurs at elevations between 200 and 300 m a.s.l., but with notable exceptions (Van Devender et al. 2000). Broadly, the distribution approaches the boundary of the Sonoran Desert as defined by Brown and Lowe (1980) and Turner (1982). However, this transition zone is patchy, with a mosaic of SDS and STS. Both *Gopherus morafkai* and *Gopherus evgoodei* occur in the more arid, desert-like ecotone-phase of STS, where limited hybridization has been observed (Edwards et al. 2015; Fig. 1). As such, we conservatively estimate the distribution of *Gopherus evgoodei* by excluding sites where *Gopherus evgoodei* and *Gopherus morafkai* come into contact (Fig. 1).

##### Natural history.

*Gopherus agassizii*, *Gopherus morafkai* and *Gopherus evgoodei* appear to have diverged roughly 5.7–5.9 Ma from a common ancestor that was potentially widespread throughout what is now the Mojave, Colorado and Sonoran desert regions (Edwards et al. 2016). *Gopherus agassizii* likely diverged first via allopatric speciation when the Bouse embayment extended northward between 8–4 Ma (Lamb et al. 1989). This waterway (now the Colorado River) created a barrier between the Sonoran and Mojave deserts. About the same time, *Gopherus morafkai* and *Gopherus evgoodei* began to segregate into tropical and arid ecosystems, possibly under a parapatric model of speciation (ecological isolation), although allopatric speciation owing to climatic change and ephemeral isolation can also explain the split. By the end of the Miocene (5.3 Ma) much of the Sonoran region was likely covered in tropical forests or desert thornscrub but orogenesis initiated the drying trend that lead to the formation of the current North American deserts. The changing environment would have created new arid niches in the northern portion of the ancestral range of the desert tortoise. This could have started the ecological divergence of the three species.

##### Microhabitat.

Ecologically, *Gopherus evgoodei* occupies hills and low mountains with at least some large boulders or rock outcrops in the TDF, and the TDF–STS ecotone. Its distribution differs from *Gopherus morafkai* by its strong association with TDF and STS, as well as its absence from SDS. Similar to *Gopherus morafkai*, *Gopherus evgoodei* often associates with slopes where rock outcrops and boulders are common. In TDF, the tortoise generally excavates burrows under already existing boulders or enters and modifies existing rock cavities. In flatter areas where boulders are not be available, it digs burrows in soil, although possibly not as extensively as its congeners. During 2012–2013 surveys in Sonora, only 9 of 44 tortoise burrows (20%) in TDF were in soil. In comparison, 56 of 87 burrows (64%) occurred in soil in STS and SDS. Local variation was not surprising. In northern Sinaloa, Vargas V (1994) reported *Gopherus evgoodei* used packrat middens, dry cacti and even burrows dug by other animals (e.g. nine-banded armadillo, *Dasypus novemcinctus*). Our observations of *Gopherus evgoodei*, as part of an ongoing radio-telemetry study near Alamos, Sonora, suggested that Goode’s Thornscrub Tortoise uses several burrows a year and exhibits strong site-tenacity, returning to familiar dens year after year (unpublished data), just like its sister-species.

##### Activity.

Presumably, tortoise activity corresponds with monsoonal rains and vegetation growth (Bury et al. 2002). Goode’s Thornscrub Tortoise is active from at least June well into November; we lack data on activity during the dry season. In Sonora, the TDF hugs the western edge of the Sierra Madre Occidental and the biome hosts extremely lush vegetation during periods of summer rainfall (July–September). During dry periods, the TDF is almost entirely leafless, but with many spectacularly blooming trees and large columnar cacti (Krizman 1972; Van Devender et al. 2000).

Little is known about daily activity patterns, reproduction, movements or forage of *Gopherus evgoodei*. Like other species of *Gopherus*, their activity relates to forage availability and ambient temperatures. Van Devender et al. (2002) reported that scat from tortoises near Alamos, Sonora contained many species of plants not found in the Sonoran Desert, suggesting differences in foraging activity and selection, although species-availability might also account for this. We observed adults to begin seasonal activity shortly in advance of the growth of forage, usually in June at the leading edge of the monsoons, and enter winter dens by sometime in December and remain underground during the dry, cool winter season (unpublished data).

##### Etymology.

The new species is a patronym, a noun in the genitive case, in recognition of Eric V. Goode, a conservationist, naturalist, and founder of the Turtle Conservancy. He has contributed generously to the conservation of this species via the preservation of land in Mexico, and he actively pursues the conservation of turtles and tortoises on a global scale. Eric sets an important precedent by complementing this taxonomic description with a tangible action that contributes to the conservation of the new species in its native habitat.

## Discussion

### Few paratypes

We designate paratypes conservatively to exclude the possibility of hybrid individuals that could confound the identity of *Gopherus evgoodei* (Edwards et al. 2010).

### Comparisons

Because of the high level of variability within all species of *Gopherus*, descriptions based on only a few individuals or individuals from few populations should be viewed cautiously. For instance, Germano (1993) calculated that female *Gopherus evgoodei* were larger than female *Gopherus agassizii* from the Mojave Desert, but our larger sample set indicates this is not the case. Legler and Vogt (2013) compared the basic proportions among *Gopherus agassizii*, *Gopherus morafkai* and *Gopherus evgoodei* (as Mojave, Sonoran and Sinaloan tortoises, respectively) and also suggested that *Gopherus evgoodei* was slightly smaller. However, analysis was also generated from a very limited dataset. Populations (sampling localities) of *Gopherus agassizii* differ widely in sizes of adults, with some populations hosting very large animals, including females, such as those in the northwestern Mojave Desert, and some hosting relatively small adult females (A. Karl; unpublished data).

Our divergence estimates for *COI* are consistent with species-level divergence in other chelonians (*Cytb*, 2.8–18.3%; Vargas-Ramirez et al. 2010). Other species of tortoises with large, continuous distributions do not exhibit the deep phylogenetic structure we observe within *Gopherus morafkai* between the Sonoran and Sinaloan lineages; for example within *Gopherus polyphemus* (Daudin) (1.5%, *ND4*; Ennen et al. 2012), *Testudo hermanni* Gmelin (1.48%, *Cytb*; Fritz et al. 2006) and *Stigmochelys pardalis* (Bell) (1.47%, *Cytb*; Fritz et al. 2010). In addition, these studies observed a range of divergences between haplogroups (intermediate haplogroups) in network analyses as opposed to the deeply bifurcating tree that typifies the matrilineal genealogy of *Gopherus morafkai*. Some species of tortoises exhibit distinct matrilines (mtDNA lineages), such as the *Testudo graeca* Linnaeus complex (mean: 3.35%, *Cytb*; Fritz et al. 2007). However, in many such cases gene flow is maintained across nuclear markers. This condition has been deemed to support the recognition of subspecies (Mashkaryan et al. 2013; Mikulicek et al. 2013). In contrast, Edwards et al. (2015) did not observe cytonuclear discordance between *Gopherus evgoodei* and *Gopherus morafkai*. The Chaco tortoise, *Chelonoidis chilensis* (Gray), of Argentina and Paraguay is perhaps the most appropriate comparison in that it has similar latitudinal range (>1,500 km), exhibits clinal variation, and occupies a variety of arid environments, including plains, deserts and semi-deserts (Fritz et al. 2012). However, the mtDNA sequence divergence in *Chelonoidis chilensis* is ~1.37% whereas the corresponding mtDNA sequence divergence between *Gopherus evgoodei* and both *Gopherus agassizii* and *Gopherus morafkai* ranges from 3.4% to 4.2%.

### Implications for conservation

Desert tortoises command a strong interest in their conservation. A distinct population segment (DPS) of *Gopherus agassizii* was federally listed in 1990 as threatened under the U.S. Endangered Species Act based on its status (USFWS 1990). *Gopherus morafkai* is considered Wildlife of Special Concern in Arizona (AGFD 2012). Mexican populations of *Gopherus* (including *Gopherus flavomarginatus*) also receive protection as threatened species (Category A “Amenazada” in NOM-059; SEMARNAT 2010).

Additional field work is necessary to assess the conservation status of *Gopherus evgoodei*, as the above summary is primarily based on observations during field work by Edwards et al. (2015) and does not include an extensive examination of population trends or threats. We lack a comprehensive understanding of its ecology and behavior. *Gopherus evgoodei* has a smaller distribution than either of its sister taxa and it occurs in some of the most threatened habitat of any of the desert tortoises (Martin and Yetman 2000). The conversion of native thornscrub to buffelgrass pasture poses the greatest threat to *Gopherus evgoodei* living in STS habitat. Conversion has specifically targeted STS in central and southern Sonora (Búrquez et al. 2002) and in TDF. Of greatest concern, this action potentially effects the operative thermal environment of the tortoise via dramatic heating. Although some parts of the STS have naturally open, desertscrub-like vegetation, the TDF and much of the STS occupied by *Gopherus evgoodei* is naturally shady in summer. The resultant thermal challenge may be especially acute in STS, which is more arid than TDF and occurs at lower elevations, and thus experiences higher temperatures.

Fortunately, successional forces can restore habitat quality for *Gopherus evgoodei* in some buffelgrass pastures in thornscrub and especially in TDF. Upon cessation of slashing and burning, secondary growth of native, woody species can quickly replace buffelgrass, and there is some evidence for this in thornscrub as well. Many local people are aware that tortoises enjoy protection and are part of nature. Their occurrence benefits society by providing employment in ecotourism and natural resource conservation. A positive trend involves the establishment and partial re-purposing of private ranches as hunting and conservation reserves throughout much of the tortoise’s distribution in Mexico. Part of the distribution of *Gopherus evgoodei* includes natural protected areas in Mexico, including Área de Protección de Flora y Fauna Sierra de Álamos-Río Cuchujaqui and certificaded área for conservation Reserva Monte Mojino in Sonora, both relatively recent institutions. If these current trends continue, environmental concerns are likely to tip the balance between pasture and native habitats somewhat in favor of tortoises, particularly if the threats to biodiversity are widely understood. However, these and other impacts on the species, such as the fragmentation of habitat, some mining activities and collection, necessitate further research that can better inform conservation and management efforts.

The recognition of *Gopherus evgoodei* reduces the area of occurrence for *Gopherus morafkai* by about 14% from roughly 171 km2 (USFWS 2015) to 147 km2. This reduction of 24,000 km2 is especially critical in Mexico where the distribution of *Gopherus morafkai* changes from 67,340 km2 (USFWS 2015) to only 43,340 km2, which is a reduction of almost 34%. By comparison, *Gopherus agassizii* occupies 83,124 km2 (Murphy et al. 2011). The IUCN considers *Gopherus agassizii* to be vulnerable to extinction (TFTSP 1996). This designation is an umbrella covering the nominate form plus *Gopherus morafkai* and *Gopherus evgoodei*. We encourage the IUCN to prepare updated assessments of the three species of *Gopherus*, as they are likely to meet the criteria for the Threatened category, particularly *Gopherus agassizii* and *Gopherus evgoodei*. Finally, all testudinids enjoy protection at least in Appendix II of CITES and *Gopherus flavomarginatus* is the only *Gopherus* with Appendix I protection. *Gopherus evgoodei* may also qualify for listing in Appendix I given its highly restricted distribution, our limited knowledge of it, threat from habitat modification and its potential to be targeted for illegal trade as a rare, charismatic animal.

## Conclusion

For decades, herpetologists have noted the distinctiveness of Mexican populations of desert tortoises in the southern part of the range of *Gopherus morafkai*, particularly where they occur in STS and TDF. Our review of recent studies sheds light on the ecology, morphology and genetics of these southern populations, which warrant species recognition of this southernmost group. Divergence estimates for *COI* and *Cytb* are consistent with species-level differences in other chelonians. *Gopherus evgoodei* primarily occurs in the state of Sonora, Mexico, extending southward into the northerly extensions of TDF in southern Sonora, northern Sinaloa, and extreme southwestern Chihuahua. The new species occurs only in STS and TDF, leaving it the smallest distribution of the three species of desert tortoises. It is important to define accurately the limits of its distribution, especially because it may occur further south in Mexico. Molecular analyses can easily diagnose all species of *Gopherus* and their hybrids (Edwards et al. 2016). Further, morphologically, *Gopherus evgoodei* is easily distinguished from *Gopherus morafkai* and *Gopherus agassizii* by several characters, among the most obvious of which is the coloration of both the shell and integument. *Gopherus evgoodei* is a dark tan to medium-brownish tortoise with a distinctly orange cast. To assess the conservation status of *Gopherus evgoodei*, additional field work is necessary as very little research on this newly described species exists and a comprehensive understanding of its ecology and behavior must be determined to inform conservation and management initiatives.

## Supplementary Material

XML Treatment for Gopherus evgoodei
